# Analysis of AgoshRNA maturation and loading into Ago2

**DOI:** 10.1371/journal.pone.0183269

**Published:** 2017-08-15

**Authors:** Alex Harwig, Zita Kruize, Zhenhuang Yang, Tobias Restle, Ben Berkhout

**Affiliations:** 1 Laboratory of Experimental Virology, Department of Medical Microbiology, Center for Infection and Immunity Amsterdam (CINIMA), Academic Medical Center, University of Amsterdam, Amsterdam, The Netherlands; 2 Institute of Molecular Medicine, Universitätsklinikum Schleswig-Holstein, University of Lübeck, Lübeck, Germany; Institut de Biologie Moleculaire et Cellulaire, FRANCE

## Abstract

The RNA interference (RNAi) pathway was recently expanded by the discovery of multiple alternative pathways for processing of natural microRNA (miRNA) and man-made short hairpin RNA (shRNA) molecules. One non-canonical pathway bypasses Dicer cleavage and requires instead processing by Argonaute2 (Ago2), which also executes the subsequent silencing step. We named these molecules AgoshRNA, which generate only a single active RNA strand and thus avoid off-target effects that can be induced by the passenger strand of a regular shRNA. Previously, we characterized AgoshRNA processing by deep sequencing and demonstrated that—after Ago2 cleavage—AgoshRNAs acquire a short 3’ tail of 1–3 A-nucleotides and are subsequently trimmed, likely by the poly(A)-specific ribonuclease (PARN). As a result, the mature single-stranded AgoshRNA may dock more stably into Ago2. Here we set out to analyze the activity of different synthetic AgoshRNA processing intermediates. Ago2 was found to bind preferentially to partially single-stranded AgoshRNA *in vitro*. In contrast, only the double-stranded AgoshRNA precursor associated with Ago2 in cells, correlating with efficient intracellular processing and reporter knockdown activity. These results suggest the presence of a cellular co-factor involved in AgoshRNA loading into Ago2 *in vivo*. We also demonstrate specific AgoshRNA loading in Ago2, but not Ago1/3/4, thus further reducing unwanted side effects.

## Introduction

**Fig 1:**
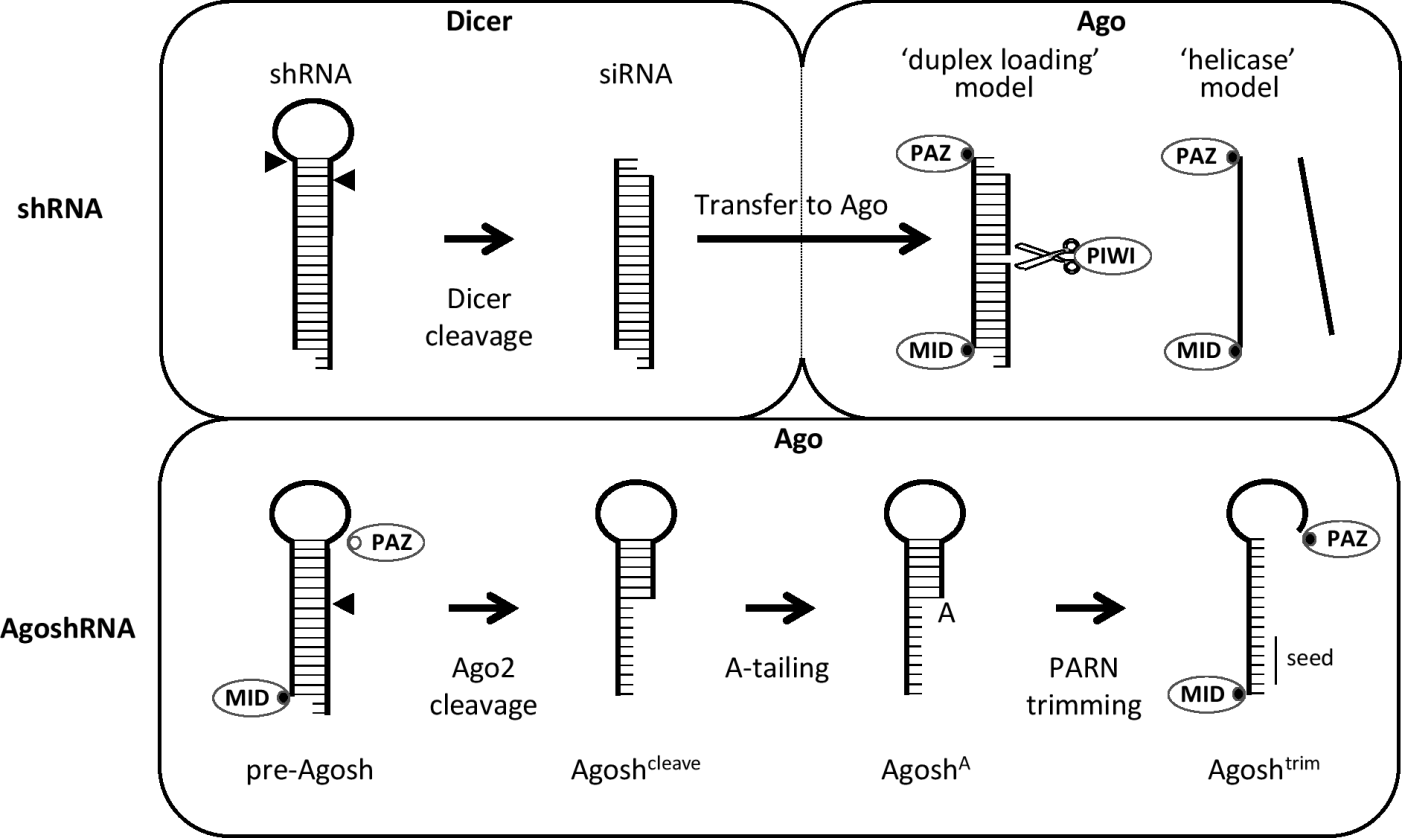
Processing and Ago2 loading of shRNA and AgoshRNA molecules. Illustration of the processing options for the Dicer-dependent shRNA and Dicer-independent AgoshRNA. Short hairpin RNAs are cleaved by Dicer resulting in a ds siRNA molecule. One of the strands is selected to be loaded into Ago2. It is however unknown whether the siRNA enters Ago as duplex, after which the passenger strand is degraded (duplex loading model), or whether one of the strands is selected prior to Ago loading by an unknown helicase (helicase model). On the other hand, the Dicer-independent AgoshRNA (and miR-451) are too small to be cleaved by Dicer and are loaded in Ago2 in their precursor form (pre-Agosh). The 5’ end can dock into the Ago2 MID-domain (closed circle), but the 3’ end is not in the vicinity of the Ago2 PAZ-domain and thus unable to dock (open circle). The hairpin is subsequently cleaved by Ago2 (Agosh^cleave^), tailed (Agosh^A^) and finally trimmed by PARN (Agosh^trim^). The resulting single-stranded molecule can dock both 5’ and 3’ ends to facilitate stable loading in Ago2.

MicroRNAs (miRNAs) and small interfering RNAs (siRNAs) are small, non-coding RNAs (∼21 nucleotides (nt) in size) that have important post-transcriptional regulatory roles by targeting messenger RNAs (mRNAs) for cleavage or translational repression [1]. miRNAs are expressed as primary miRNA (pri-miRNA) transcripts and subsequently processed. The nuclear Microprocessor complex, composed of the RNAse III-like endonuclease Drosha and its double stranded RNA (dsRNA)-binding partner DGCR8, cleaves the stem-loop structure embedded in the pri-miRNA, and releases a smaller ~70 nt precursor miRNA (pre-miRNA) hairpin with a 2-nt 3’ overhang [2, 3]. This pre-miRNA is subsequently exported from the nucleus to the cytoplasm, where the RNAse III-like Dicer endonuclease will remove the terminal loop to generate a duplex of ~20–24 base pairs (bp) [4, 5]. RNAi can also be induced by man-made constructs containing RNA polymerase III gene cassettes that express a short transcript with the characteristics of a pre-miRNA [6–8]. Such short hairpin RNA (shRNA) molecules (Fig 1, top) skip Microprocessor processing and enter the RNAi pathway at the Dicer processing step, resulting in the generation of a small interfering RNA (siRNA) duplex. Finally, the miRNA or siRNA duplex is incorporated into the RNA-induced silencing complex (RISC) by association with the Argonaute (Ago) protein [9, 10].

It was always assumed that depending on the thermodynamic properties of the miRNA duplex, one strand will be preferentially selected as mature miRNA and guide the RISC complex to complementary mRNA targets [11, 12]. However, recently it has been suggested that Dicer-TRBP presents the cleaved miRNA duplex to Ago in a manner that determines the strand specificity [13]. This is in contrast to previous studies showing that Dicer or its partner proteins are not essential for assembly of human Ago2-RISC [14–16]. Another possibility would be that a chaperone-like system, as seen in Drosophila, assists with loading of human Ago2 [17]. Furthermore, recent biochemical and structural studies indicate that conformational changes in Ago2 proteins are likely the main driving force of the small RNA duplex separation [18–20]. Successful anchoring of the 5’ phosphate in the MID domain would trigger loading of the rest of the siRNA duplex, leading to passenger strand ejection and mature RISC formation. However, the molecular mechanism of how the passenger strand is omitted from Ago still remains relatively elusive. There are two models for loading of a small RNA duplex into Ago2 to form RISC, the ‘duplex-loading model’ and the ‘helicase model’ (Fig 1, top). The helicase model was originally most popular because several helicases have been implicated in RISC formation [21–24], and the asymmetry rule is consistent with unwinding of the small RNA duplex from the ‘easier-to-unwind’ end by a helicase [11, 12]. In this model, Ago2 would thus exclusively load ssRNA. However, the putative helicase has not yet been identified. The current, alternative duplex model assumes that Ago2 receives the dsRNA and cleaves the passenger strand much like it would cleave a target mRNA, followed by passenger strand release and degradation [9, 10, 25]. However, it remains unclear how Ago can unwind the nicked duplex of a perfectly base paired RNA duplex as it possesses only weak strand-dissociating activity [26].

The preferential loading based on thermodynamic properties of the duplex means that both strands of the siRNA duplex can be loaded into Ago to form an active RISC, possibly leading to off-target effects. Recently, we described shRNA molecules that skip the Dicer processing step and instead are processed by Ago2. The unique slicing ability of Ago2, results in a single active siRNA strand (Fig 1, bottom). These so-called AgoshRNAs resemble the natural miR-451 molecule [27, 28] in being typically shorter (17–19 bp) than regular shRNAs and miRNAs, and having a small loop of 3–5 nt [29, 30].

Previously, we characterized AgoshRNA processing by deep sequence analysis of Ago2-bound intermediates present in cells [31]. A model was proposed (Fig 1, bottom) in which the pre-AgoshRNA precursor is loaded into Ago2, thereby docking the 5’ phosphate in the MID domain (closed circle). However, whereas the active strand of a miRNA would thread through the Ago groove, the ds pre-AgoshRNA hairpin cannot, thus leaving the 3’ binding pocket in the PAZ domain unoccupied (open circle). Ago2 cleaves the 3’ strand of the pre-AgoshRNA hairpin between the 10^th^ and 11^th^ bp, yielding a ~30 nt partially dsRNA (Fig 1, Agosh^cleave^). We show this product in Fig 1 as partially base paired. However, maximally 9 base pairs can be formed and some of these molecules may be unwound within Ago2. Based on available structural data [19], such unwinding would be essential to make the 3’-end accessible for subsequent processing. Subsequent 3’-Adenylation (Fig 1, Agosh^A^) [31], similar to 3’-Uridylation in miR-451 [27], labels the RNA as a target for poly(A)-specific ribonuclease (PARN), which will trim the 3’ end to create the active ssRNA species (Fig 1, Agosh^trim^). The processed AgoshRNA is a ssRNA that can properly dock the 5’ and 3’ ends into Ago [31].

In this study, we investigated the interaction of the AgoshRNA processing intermediates with Ago and probed the generation of functionally active RISC complexes. Attempts to describe sequence-activity relationships using mutant gene constructs are troubled by the multiple effects imposed at the level of transcription, processing, transcript stability and AgoshRNA activity. To avoid such complications, we made synthetic AgoshRNA molecules to score the activity of several processing intermediates in appropriate *in vitro* and in cell culture-based assays [31]. Fig 1 illustrates the four intermediates that we tested: the precursor pre-Agosh, Ago2 cleavage to Agosh^cleave^, 3’ adenylation to form Agosh^A^ and trimming to generate Agosh^trim^.

We demonstrate that the partially dsRNA molecules with a 3’ unpaired end (Agosh^A^ and Agosh^trim^) interact most efficiently with Ago in cells, whereas in cells Ago2 exclusively loads the ds precursor pre-Agosh. The pre-Agosh is exclusively processed in cells and is the only RNA species that is active in knock down assays. These results suggest that a cellular co-factor is needed to facilitate Ago2 loading *in vivo*, after which the small RNA is processed into a ss guide strand for a more stable interaction with Ago2, as was measured *in vitro*. Furthermore, our results suggest that AgoshRNA molecules interact preferentially with Ago2 and not with the Ago1/3/4 complexes.

## Materials and methods

### Cell culture, transfection and RNA isolation

Human Embryonic Kidney (HEK) 293T cells were cultured as monolayer in Dulbecco’s Modified Eagle’s Medium (DMEM, Gibco) supplemented with 10% (v/v) heat-inactivated Fetal Calf Serum (FCS) and 1% (v/v) non-essential amino acids (Gibco) at 37°C and 5% CO2. For the transfections, HEK 293T cells (5 x 10^6^ cells) were cultured in 25 cm^2^ flasks and transfected with 50 nM synthetic AgoshRNA using lipofectamin2000 (Life technologies). For co-immunoprecipitation, 50 nM synthetic AgoshRNA was co-transfected with 500 ng FLAG-tagged Ago-expression plasmid. RNA was isolated from transfected HEK 293T cells using the mirVana™ miRNA Isolation Kit (Ambion) according to the manufacturer's instructions for total RNA isolation. RNA concentrations were measured with the NanoDrop 2000 (ThermoFisher Scientific).

### Synthetic AgoshRNA molecules

The AgoshRNA-based molecules used in this study were ordered as synthetic RNA oligonucleotides (IDT). These AgoshRNA sequences are based on the anti-HIV Gag5 shRNA [32] and represent the different AgoshRNA processing products detected by deep sequencing [31]. Exact sequences are presented in Table 1.

**Table 1:** Sequence of AgoshRNA-based molecules.

Construct		Sequence	nt
pre-Agosh	A	5’-AGCUGUCAUCAUUUCUUCUCAAGAAGAAGAAAUGAUGACAGCC-3’	43
Agosh^cleave^	B	5’-AGCUGUCAUCAUUUCUUCUCAAGAAGAAGAAAU-3’	33
Agosh^A^	C^A^	5’-AGCUGUCAUCAUUUCUUCUCAAGAAGAAGAAAUA-3’	34
Agosh^U^	C^U^	5’-AGCUGUCAUCAUUUCUUCUCAAGAAGAAGAAAUU-3’	34
Agosh^trim^	D	5’-AGCUGUCAUCAUUUCUUCUCAAGAA-3’	25

### Fluorescence measurements of AgoshRNA binding to Ago2

Affinities of the different AgoshRNA molecules for Ago2 were measured as previously described [33]. In brief, full length Ago2 was purified as a GST fusion protein from Escherichia coli BL21(DE3) harboring the pET41b(+) plasmid coding for Ago2. SDS-PAGE gel analysis of this recombinant hAgo2 preparation is presented in S1 Fig. This purified Ago2 was bound to a fluorescently labeled ds-siRNA (FAM-as2b/s2b) in 10 mM Tris/HCl (pH 7.4) and 100 mM KCl. The guide strand of the siRNA (FAM-as2b: 5'-uag agg uac gug cTg agg cTT; DNA nt’s in capitals) was labeled with the FAM fluorescein derivative coupled to T14 and hybridized with the corresponding passenger strand (s2b: 5'-uag agg uac gug cug agg cTT; DNA nt’s in capitals). Titrations were performed with increasing concentrations of AgoshRNA (up to 600 nM) and displacement of the labeled ds-siRNA was measured. To monitor the fluorescence change upon displacement of the labeled siRNA from Ago2, the samples were excited at 492 nm, and the emission intensity was measured at 520 nm on a FluoroMax-3 spectrofluorometer. This assay scores a change in fluorescence exclusively for properly bound substrates [33]. The changes in fluorescence may seem rather low, but that is because the values were normalized to 1, and the observed 15–20% change is within the range of previous studies [33].

### Plasmid constructs

The wild-type AGO2 protein was expressed from the pIRESneo-Flag/HA-AGO2 vector that was kindly provided by Y. Tomari [34]. The Ago-expression plasmids pIRESneo-FLAG/HA Ago1 (#10820), pIRESneo-FLAG/HA Ago2 (#10822), pIRESneo-FLAG/HA Ago3 (#10823) and pIRESneo-FLAG/HA Ago4 (#10824) were obtained from Addgene (Cambridge, MA, USA).

### Ago2 immunoprecipitation and RNA isolation

Ago2-FLAG immunoprecipitation and isolation of the bound RNA molecules has been described previously [31]. Briefly, HEK 293T were co-transfected with the synthetic AgoshRNA molecule and Ago2-FLAG plasmid and the cells were washed several times with cold PBS after 48 hours. Lysis of the cells was achieved by incubation with IsoB-NP40 (10 mM Tris-HCl (pH 7.9), 150 mM NaCl, 1.5 mM MgCl_2_ and 1% NP-40) for 20 minutes on ice. To clear cell debris, the lysates were centrifuged for 10 minutes at 13,000 x g at 4°C. The supernatant was incubated overnight with 75 μL of anti-FLAG M2 agarose beads (Sigma-Aldrich) at 4°C with constant rotation. The supernatant (depleted fraction) was separated from the beads (bound fraction) by centrifugation for 30 seconds at 7,000 x g, after which the depleted fraction was transferred to a new 1.5 ml tube and kept on ice until use. The beads were washed three times in NET-1 buffer (50 mM Tris-HCl (pH 7.5), 150 mM NaCl, 2.5% Tween 20). When indicated, a subset of the bound and unbound fractions was dissolved in 2x Laemmli buffer for subsequent western blot analysis. Small RNAs associated with Ago2 (and those in the unbound fraction) were isolated by acid-phenol: chloroform extraction followed by ethanol precipitation. RNA concentrations were measured with the NanoDrop 2000 (Thermo Scientific) and subsequently analyzed on northern blot.

### Northern blot

Isolated RNA (5 μg), synthetic AgoshRNA (100 pmol) and Ago-FLAG immunoprecipitated RNA (1 μg) were analyzed by northern blotting. The RNA samples were denatured for 5 minutes at 95°C before electrophoresis on a 15% denaturing polyacrylamide gel (precast Novex TBU gel, Invitrogen). To check for equal sample loading, the 5.8S rRNA and tRNA bands were visualized by ethidium bromide staining (2 μg/ml) for 20 minutes. The RNA samples were electrotransferred to a positively charged nylon membrane (Boehringer Mannheim, GmbH, Mannheim, Germany) and crosslinked to the membrane using UV light at a wavelength of 254 nm (1200 μJ × 100) in the Stratalinker UV Crosslinker 1800 (Agilent Technologies). LNA oligonucleotide probes were 5’ end labeled with the KinaseMax™kit (Ambion, Carlsbad, CA) in the presence of 1 μL [γ-^32^P]ATP (0.37 MBq/μL, PerkinElmer) according to manufacturer’s instructions. To remove unincorporated nucleotides, the LNA probes were purified using Sephadex G-25 spin columns (GE Healthcare) according to manufacturer’s protocol. Hybridization was performed overnight at 42°C with labeled LNA oligonucleotides in 10 ml ULTRAhyb hybridization buffer (Ambion) according to the manufacturer's instructions. To detect the 5’ strand the Gag5 sense probe was used (LNA positions are underlined) 5’-ATGCTGTCATCATTTCTTC-3’ and to detect the 3’ strand the Gag5 antisense probe was used (LNA positions are underlined) 5’-GAAGAAATGATGACAGCAT-3’. After overnight hybridization the membranes were washed 3–4 times for 5 minutes at 42°C in 10 ml 2 × SSC/0.1% SDS (low stringency) and twice for 15 minutes at 42°C in 10 ml 0.1 × SSC/0.1% SDS (high stringency). Signals were detected and quantified using a phosphorimager (Typhoon Phosphorimager).

### SDS-PAGE western blot

For SDS-PAGE western blot the samples obtained in the Ago2 pull down assays were mixed with an appropriate amount of loading dye (25 mM Tris, 192 mM Glycine, 20% v/v glycerol, 4% m/v SDS, 0.1% v/v bromophenol blue in milli-Q water). The samples were denatured at 95°C for 5 minutes before electrophoresis on a Novex 6% Tris-glycine gel (Invitrogen). The samples were transferred to an Immobilin-P Transfer Membrane at 25V for 50 minutes. The membrane was then blocked for 60 minutes in 5% milk powder in PBS. The membrane was washed with 0.1% milk-PBS and incubated overnight at 4°C in 10 ml 0.1% milk-PBS with anti-FLAG primary antibody (1:5000) or alpha-actin (1:5000) with slow orbital mixing. After overnight incubation, the membrane was washed 3–4 times for 5 minutes with 0.1% milk-PBS and then incubated for at least one hour with slow orbital mixing in 10 ml 0.1% milk-PBS with goat anti-mouse secondary antibody (1:5000). The membrane was washed 2 times for 5 minutes in PBS-Tween, 2 times for 5 minutes in PBS and 2 times for 5 minutes in deionized H_2_O. The Western Lightning ECL system (PerkinElmer Life Sciences) was used for luminometric detection with the LAS4000.

### Cloning of AgoshRNAs

The pre-Agosh RNA samples obtained from the Ago2 immunoprecipitation were cloned and sequenced. For this purpose, the small RNAs were 3’ polyadenylated using the A-Plus Poly(A) Polymerase Tailing Kit (Epicentre Biotechnologies) according to manufacturer’s protocol. The reaction was stopped by acid-phenol:chloroform extraction followed by ethanol precipitation. Subsequently, the polyadenylated RNAs were reverse transcribed into cDNA, using the ThermoScript RT-PCR System for first-strand cDNA synthesis (ThermoFisher Scientific), followed by PCR amplification. PCR products were separated on size via agarose gel electrophoresis, purified and subsequently used for TOPO-TA cloning (TOPO-TA cloning kit, Life Technologies). TA clones were sequenced with the T7 and m13RP primer using the BigDye Terminator v1.1 Cycle Sequencing Kit (Applied Biosystems).

### Dual luciferase assay

HEK 293T were co-transfected with 100 ng of firefly luciferase expression plasmid (Gag5 sense or antisense), 0.5 ng of renilla luciferase expression plasmid (pRL-CMV) and 5 μL of AgoshRNA expression construct of different dilutions (10–0.001 μM). The pRL plasmid served as an internal control for cell viability and transfection efficiency and pBluescript SK- (pBS) (Promega) was added to obtain equal DNA concentrations. Two days post-transfection, firefly and renilla luciferase activities were assessed using the Dual-Luciferase Reporter Assay System (Promega) according to the manufacturer's protocol.

## Results

### *In vitro* binding of AgoshRNA processing intermediates to Ago2

**Fig 2:**
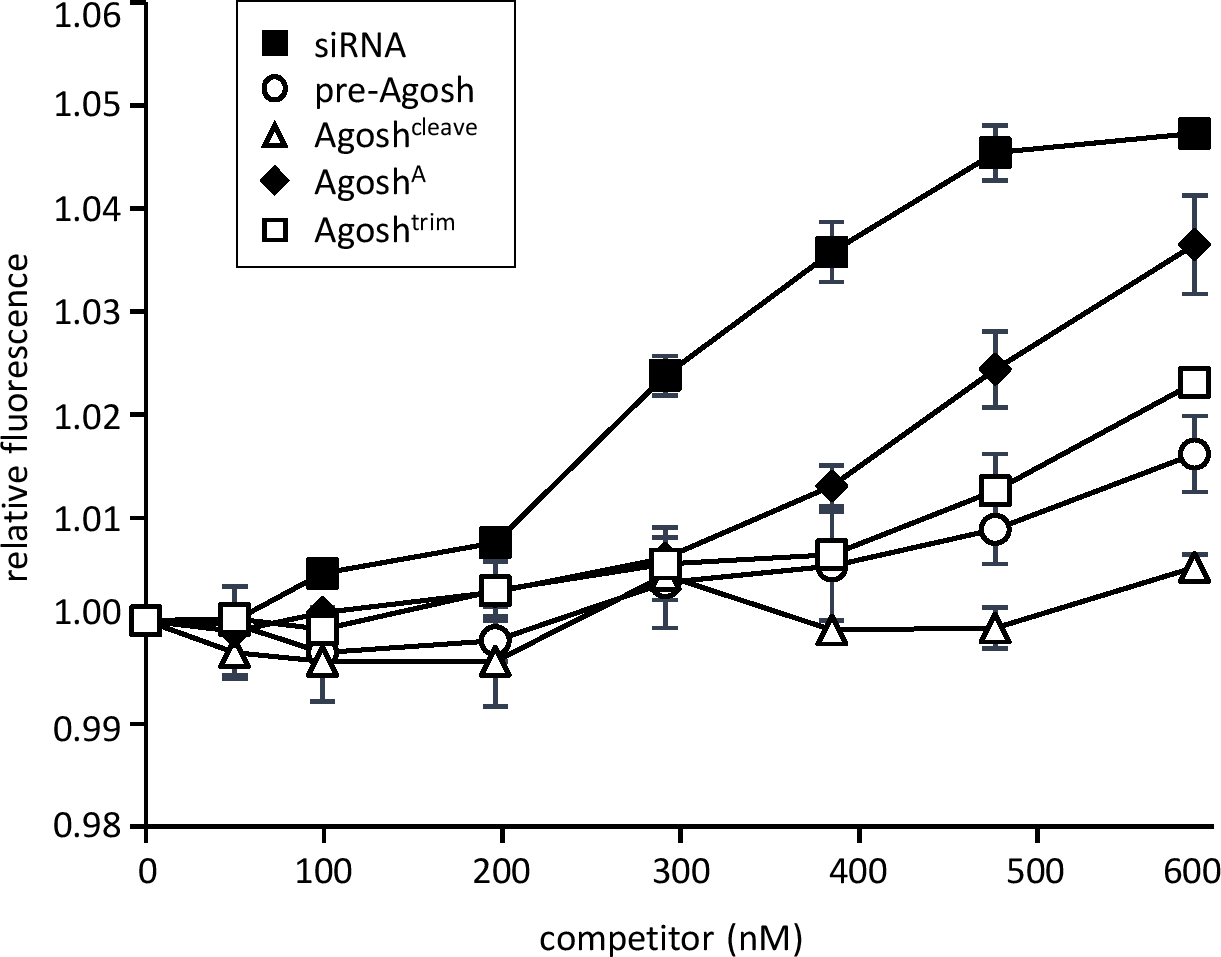
*In vitro* binding of the AgoshRNA-based molecules to Ago2. The complex forming of hAgo2 and AgoshRNAs was measured by equilibrium fluorescence titration. FAM-labeled substrate (20nM FAM-as2b/s2b) was used as the ds siRNA with known affinity for hAgo2 (black square). Relative fluorescence (y-axis) was scored for all constructs and the x-axis shows the concentration of competitor (AgoshRNAs) added in nM. The graph shows the mean values and standard deviations of two independent experiments.

Synthetic AgoshRNA molecules were developed based on the distinct AgoshRNA processing intermediates shown in Fig 1: pre-Agosh, Agosh^cleave^, Agosh^A^ and Agosh^trim^. These AgoshRNA-forms were first tested for their ability to interact with recombinant human Ago2 (Ago2) *in vitro* by means of equilibrium fluorescence titration [33]. We pre-formed an Ago2 complex with a FAM-labeled siRNA duplex (as2b-FAM/s2b) and added increasing concentrations of the unlabeled AgoshRNA molecules, followed by monitoring of changes in the fluorescent signal (Fig 2). The ds siRNA control was an efficient competitor, consistent with previous results [33]. All forms of the synthetic AgoshRNA-set demonstrated binding activity, but Agosh^A^ was the best competitor, followed by Agosh^trim^, pre-Agosh and Agosh^cleave^.

### Exclusive cellular processing of the pre-AgoshRNA precursor

**Fig 3:**
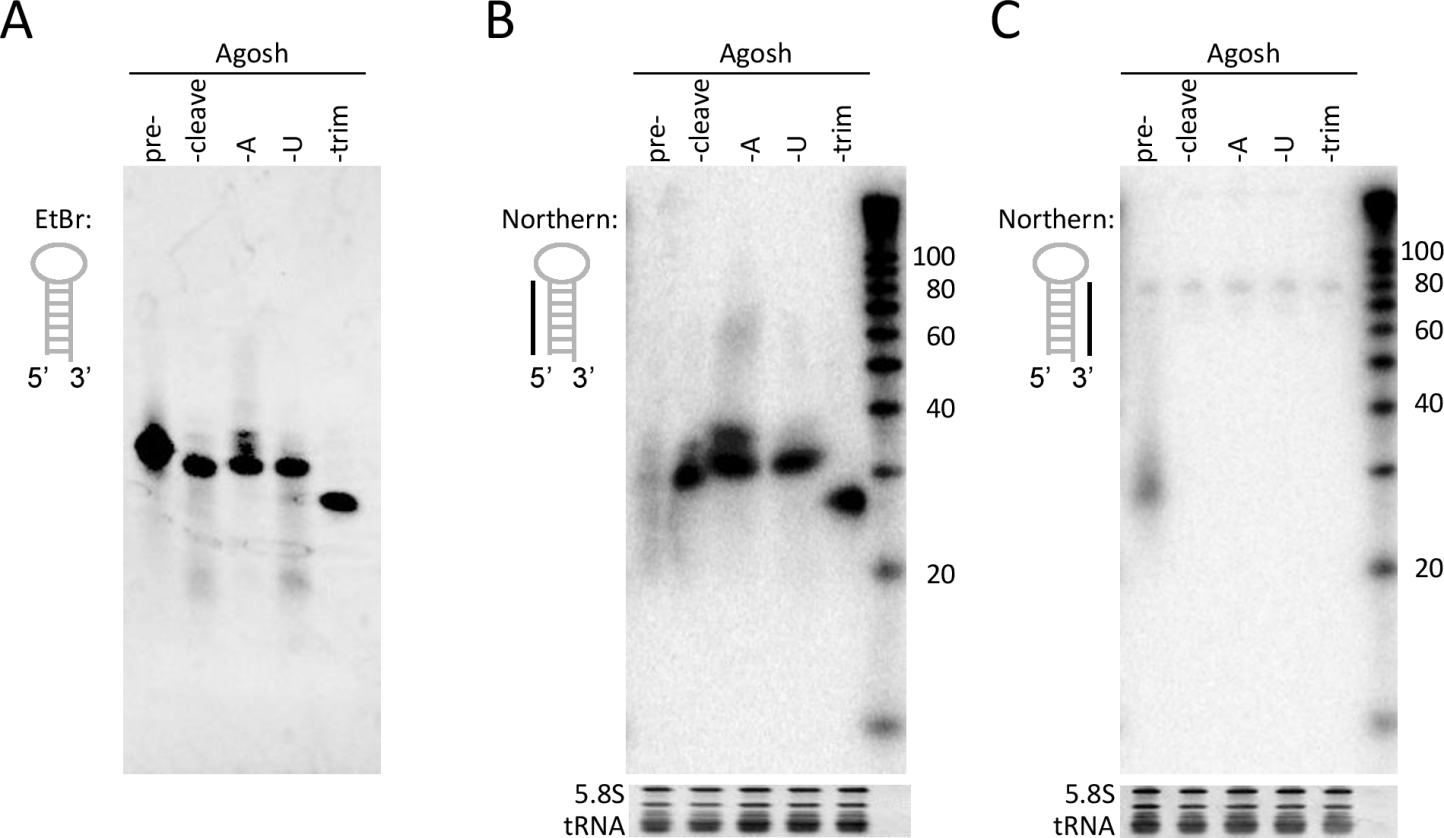
*In vivo* processing of the synthetic AgoshRNA molecules. **(A)** 100 pmol of the synthetic RNAs was analyzed on a 15% polyacrylamide gel and stained with ethidium bromide. **(B)** HEK 293T were transfected with 100 pmol of the synthetic RNA molecules. Total cellular RNA was isolated after 48 h and analyzed by northern blot. An LNA probe targeting the 5’ side of the hairpin was used. A size marker is included on the right hand side. Ethidium bromide staining of 5.8S rRNA and tRNAs is included below the blot as loading control. **(C)** As B, but now using an LNA probe targeting the 3’ side of the hairpin.

Next, we initiated cell culture based tests for the set of synthetic AgoshRNA molecules. Stability and potential processing of the synthetic AgoshRNAs were assessed by RNA-transfection into 293T cells. We also included an Agosh^cleave^ variant with a 3’ U-extension (Agosh^U^) to resemble the miR-451 processing intermediate [27]. To do so, total cellular RNA was isolated at 48 hours post-transfection and the synthetic RNAs were analyzed by gel electrophoresis and northern blotting. The input synthetic RNA was visualized by ethidium bromide staining (Fig 3A), displaying bands of the expected length: 43 nt for pre-Agosh, 33 nt for Agosh^cleave^, 34 nt for Agosh^A^ and Agosh^U^, and 25 nt for Agosh^trim^. Cellular RNA was blotted and we used probes to detect either the 5’ or 3’ side of the hairpin (Fig 3B and 3C, respectively). Only the pre-Agosh showed signs of intracellular processing by disappearance of the 43 nt input band, yielding an array of shorter products. All processing products (Agosh^cleave^, Agosh^A^, Agosh^U^, and Agosh^trim^) show the unaffected input RNA with the 5’ probe. This exclusive processing of pre-Agosh yielded multiple products, which appeared as a smear of differently sized RNA molecules. The 3’ probe showed a similar array of shortened RNA products. This pattern was reproducibly observed and is not due to RNA degradation in the sample, as was confirmed by ethidium bromide staining of intact cellular tRNAs and 5.8S rRNA. As expected, the 3’ probe was not able to detect the processed AgoshRNA forms due to insufficient sequence complementary with the truncated 3’ arm.

### Exclusive reporter knockdown by the AgoshRNA precursor

**Fig 4:**
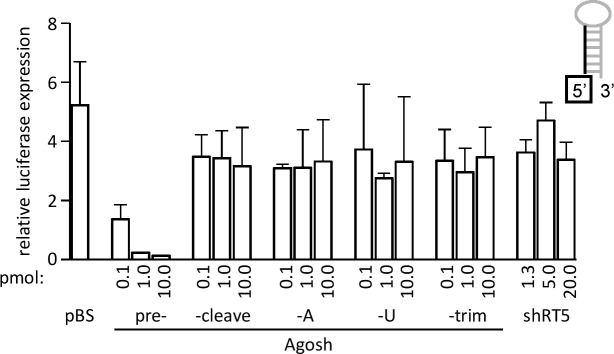
Reporter knock down activity of the AgoshRNA molecules. The knockdown activity of the guide strand of the synthetic RNA molecules was determined by co-transfection with the corresponding luciferase reporter in HEK 293T cells. An unrelated shRNA (shRT5) and the ‘empty’ vector pBluescript served as negative control. We performed three independent transfections, each in duplicate, and standard deviations were calculated.

The silencing activity of the AgoshRNA set was tested on a luciferase reporter that contains a perfectly complementary target sequence. We co-transfected the luciferase construct with increasing amounts of the synthetic AgoshRNA (0.1–10 pmol) into HEK 293T cells. Co-transfection with the pBluescript plasmid (pBS) or an unrelated shRNA expressing plasmid (shRT5) served as negative controls. We scored high luciferase knockdown activity for the pre-Agosh (Fig 4). All processed AgoshRNA forms did not show any luciferase knockdown activity.

### Preferential loading of the AgoshRNA precursor in Ago2

**Fig 5:**
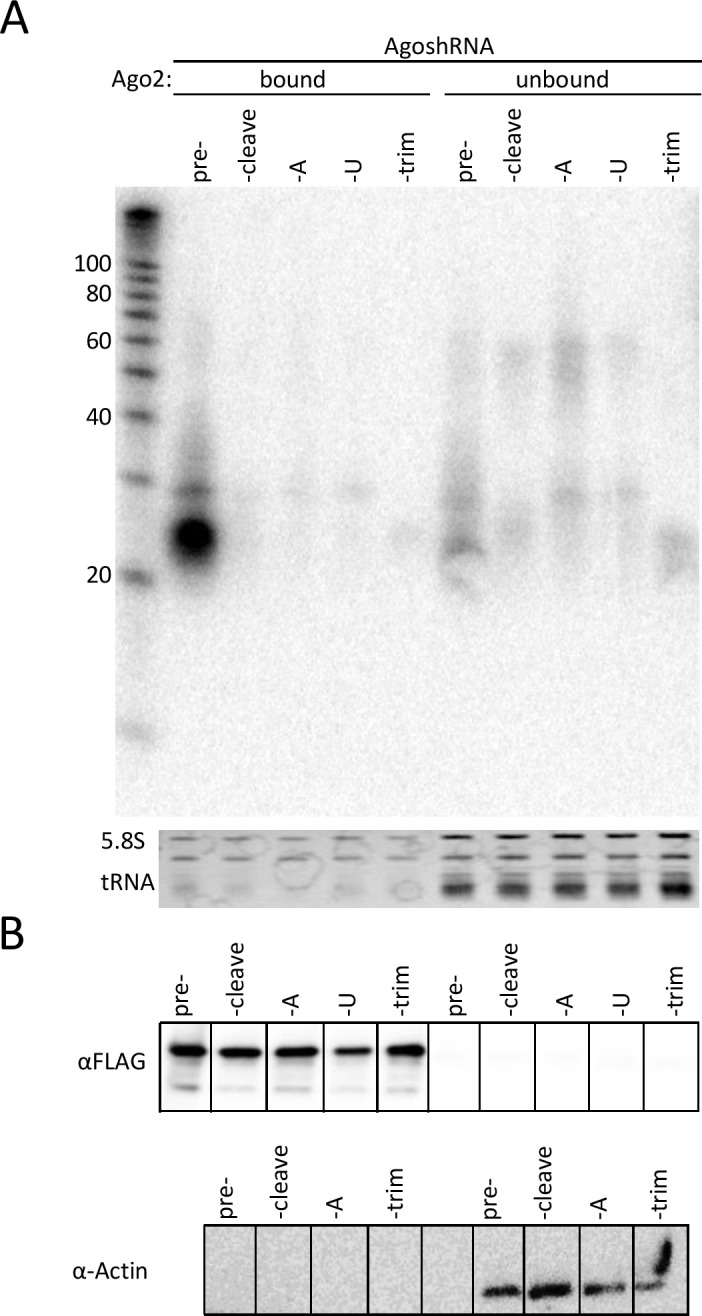
*In vivo* complex formation of the AgoshRNA set and Ago2. **(A)** Northern blot analysis of immunoprecipitated AgoshRNAs with Ago2-FLAG. Both bound and depleted (unbound) fractions are shown for all synthetic RNA molecules. A size marker is included on the left hand side. Ethidium bromide staining of 5.8S rRNA and tRNAs was included as loading control below the blots. **(B)** Western blot analysis of the Ago2-bound and unbound fractions using antibodies against FLAG and α-Actin.

Next, we set out to investigate Ago2 loading of the AgoshRNA set in cell culture based systems. Co-transfection of the synthetic RNAs and an expression plasmid encoding FLAG-tagged Ago2 was performed in HEK293T. Cells were lysed after 48 hours and Ago2 was immunoprecipitated using an anti-FLAG antibody. The samples were subjected to stringent washing and Ago2-associated RNAs (bound fraction) were separated from the unbound fraction. The bound and unbound fractions were analyzed by northern blot with an LNA probe complementary to the 5’ side of the hairpin (Fig 5A). A prominent ~24 nt fragment and minor ~30 nt fragments were detected, but exclusively for the pre-Agosh molecule. The processed AgoshRNA forms (Agosh^cleave^, Agosh^A^ or Agosh^U^, and Agosh^trim^) were not enriched in the Ago2-bound fraction and only weak signals were present in the unbound fractions. The weak RNA signal in the unbound fraction could relate to the RNA isolation method as these RNAs are abundantly present in total RNA (see Fig 2B). Capture of the FLAG-tagged Ago2 complexes was confirmed by the appearance of a ~160 kDa product on western blot (Fig 5B; upper panel). This molecular mass resembles the size of loaded RISC [35]. An equal protein content of the unbound fractions was confirmed by western blotting for α-actin (Fig 5B; lower panel).

We also analyzed the RNA contents of the immunoprecipitated complexes of the pre-AgoshRNA by RT-PCR, TA-cloning and sequencing [29]. Of the eleven TA-clones obtained, the sequences correspond to Agosh^A^ (1x), Agosh^trim^ (7x), or intermediates (3x) that suggest gradual trimming from the 3’ end of Agosh^A^ (Table 2). The size of these fragments correlates with that of Ago2-bound RNA fragments detected on northern blot (Fig 5A). No RNA fragments corresponding to the pre-Agosh or the initial processing product Agosh^cleave^ were detected. Thus, we observed a perfect correlation between exclusive loading of the pre-Agosh molecule (Fig 5A), intracellular processing (Fig 3B) and reporter knockdown activity (Fig 4). These results are consistent with the idea that Ago2 loading and subsequent processing is required for AgoshRNA activity.

**Table 2:** Sequences of Ago2-associated RNA.

RNA product	sequence	clones
pre-Agosh	AGCUGUCAUCAUUUCUUCUCAAGAAGAAGAAAUGAUGACAGCC	0x
Agosh^cleave^	AGCUGUCAUCAUUUCUUCUCAAGAAGAAGAAAU	0x
Agosh^A^	AGCUGUCAUCAUUUCUUCUCAAGAAGAAGAAAUA	1x
Agosh^trim^	AGCUGUCAUCAUUUCUUCUCAAGAA	7x
30 nt intermediate	AGCUGUCAUCAUUUCUUCUCAAGAAGAAGA	2x
28 nt intermediate	AGCUGUCAUCAUUUCUUCUCAAGAAGAA	1x

### AgoshRNA binds selectively to Ago2 and not Ago1/3/4

**Fig 6:**
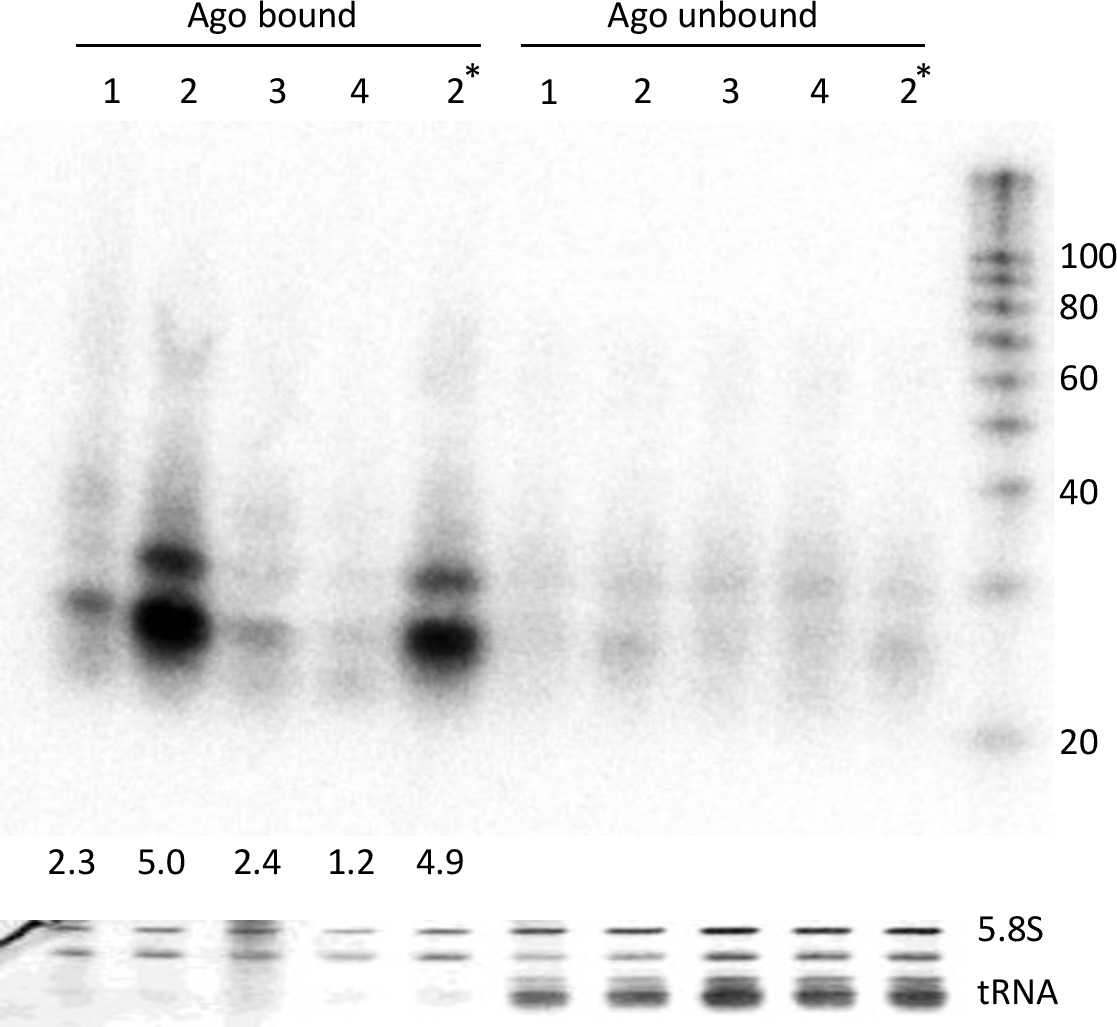
*In vivo* complex formation of the AgoshRNA set with Ago1-4 proteins. Northern blot analysis of immunoprecipitated pre-Agosh with all four human Ago proteins. Both the bound and depleted (unbound) fractions are shown. A size marker is included on the right hand side. Ago2* was expressed from a different vector as the other four Argonaute proteins. Fold enrichment of bound over unbound is indicated underneath lane. Ethidium bromide staining of 5.8S rRNA and tRNAs is shown as loading control below the blot.

Human cells encode four Argonaute proteins: Ago1-4 [36]. All Ago proteins can load small RNAs, but only Ago2 has endonuclease activity [37]. Ago2-selective loading has also been reported for some molecules, including the Dicer-independent miR-451 [38]. We therefore chose to probe AgoshRNA loading in the four Ago proteins, also because interaction with any of the alternative Ago proteins could possibly trigger off target effects [39, 40]. We explored Ago1-4 loading by co-transfection of the pre-Agosh with plasmids encoding one of the Ago forms as FLAG-tagged protein. The Ago proteins were immunoprecipitated with anti-FLAG antibody after 2 days and the associated RNA was extracted and analyzed on northern blot (Fig 6). Equal amounts of Ago-specific immunoprecipitated RNA were loaded to correct for expression differences of the various Ago proteins. The Ago2-FLAG expression plasmid used in the previous experiments was also included (labeled 2* in Fig 6). Immunoprecipitation of Ago2 yielded two RNA products, a prominent band of ~24 nt and a minor ~30 nt fragment. These products were enriched at least 5x in the bound versus the unbound fraction. The pattern and abundance of Ago2-bound RNA products agree with the immunoprecipitation results shown in Fig 5A. Most importantly, Ago1 and 3 yielded a minor 2x RNA enrichment over the unbound fraction and Ago4 did not show any RNA enrichment, suggesting preferential Ago2 loading of AgoshRNA molecules, similar to the properties of miR-451. However, this assay does not allow a more quantitative analysis as the Ago3/4 expression level can be much reduced compared to that of Ago1/ 2 [41].

## Discussion

Novel AgoshRNA designs form a promising class of RNAi-based therapeutics that do not require Dicer cleavage and instead use Ago2-slicer activity to generate a guide RNA that targets a complementary mRNA for degradation. The major benefit of the AgoshRNA design over regular shRNAs is that off-target effects induced by the passenger strand are avoided. Earlier studies pinpointed to the stem length as a critical element for Ago2-mediated processing to avoid Dicer-processing [30]. Recently, several novel details about AgoshRNA processing were uncovered by deep sequencing analysis, such as 3’ A-tailing and trimming [31]. In the present study we set out to obtain more insights about AgoshRNA processing by Ago2 and the activity of the processing intermediates using synthetic RNA templates in *in vitro* and cell culture based experiments. We observed that Ago2 specifically loads the ds precursor AgoshRNA (pre-Agosh) in cells, which explains why it is exclusively processed inside the cell and active in reporter mRNA knockdown.

In literature, two models exist to explain how small RNAs are incorporated into Ago (Fig 1: ‘duplex loading model’ versus ‘helicase model’). The former model is currently more widely accepted [42]. However, the loading pathway for the smaller AgoshRNAs is completely unknown. Our results seem to add conflicting evidence in favor of these alternative models for AgoshRNA. The *in vitro* results imply that Ago2 favorably loads partially ds/ss Agosh^A^ and ss Agosh^trim^ intermediates over the ds pre-Agosh precursor (Fig 2). These results seem to support the helicase model in which Ago needs to—at least partially—unwind the guide strand to initiate the loading process. Surprisingly, the partially ss Agosh^cleave^, which differs from Agosh^A^ in only a single nt, is a very bad competitor for Ago binding (Fig 2). However, the A-addition creates a free 3’ end for the Agosh^A^ molecule, possibly facilitating easier 3’ end loading in the 3’ binding pocket of the PAZ-domain. In striking contrast, the cell culture-based studies indicate exclusive Ago2 loading of the ds pre-Agosh and not all the partially ss intermediates (Fig 5). This specific Ago2-association with the ds pre-Agosh correlates with exclusive *in vivo* processing (Fig 3) and knockdown activity (Fig 4). These combined *in vivo* results strongly support the duplex loading model applies also to AgoshRNA.

The discrepancy between the *in vitro* and cell culture results could be explained by the involvement of cellular co-factors for RNA loading in Ago2 that are lacking in the *in vitro* assays with recombinant Ago2. For instance, recent studies indicate that the Dicer-TRBP complex is required to present the correct guide strand for Ago2 loading to form an active RISC-complex [13, 43], while others show that Dicer or its partner proteins are not essential for assembly of RISC [14–16]. We previously demonstrated AgoshRNA activity in HCT116 cells that are deficient for Dicer cleavage [30], but this cleavage-deficient Dicer [44] may still be able to interact with the pre-miRNA and subsequently present the hairpin to Ago. Dicer may inspect the small ds hairpin of the pre-Agosh, which is not cleaved because the stem is too short [45–47]. The uncleaved AgoshRNA may subsequently be presented to Ago, which can initially dock the 5’ end in the MID-domain, followed by processing of this short duplex into a ss RNA molecule. In this study we use a functional minimal RISC consisting of only Ago without co-factors, which is still able to bind and cleave target RNAs [33]. This *in vitro* Ago-only system is comparable to others [48–50], and is commonly used as a tool to investigate Ago-binding kinetics of small RNA. However, the loading of ss small RNA seems not to be possible in cells. This is likely caused by co-factors that regulate proper loading of RISC in vivo [13, 17].

We also show that AgoshRNAs are preferentially loaded in Ago2 over Ago1/3/4 (Fig 5 and Fig 6), thus resembling miR-451 [38]. This preferential loading correlates with disappearance of the pre-Agosh in the total RNA fraction (Fig 3) and RISC-mediated silencing activity (Fig 4). These combined results argue that only the ds preAgosh can be used *in vivo* to create a functional RISC. Implication for future gene therapy will be that the effector AgoshRNA molecule should be either a ds RNA or an expressed small hairpin.

In this study we investigated the processing intermediates of the novel AgoshRNA design in detail. We demonstrate that the ds precursor AgoshRNA (pre-Agosh) is selected for loading into Ago2 in cells to form an active RISC, while the partially-ds/ss intermediates are not functional. We also show that this selection step is circumvented by recombinant Ago2 *in vitro* as the partially-ds/ss AgoshRNA intermediates (Agosh^A^ and Agosh^cleave^) bind much better than pre-Agosh. We thus conclude that the AgoshRNA hairpin likely requires co-factors for Ago2 loading *in vivo*. Ago2-mediated processing of the ds RNA into a ss species subsequently facilitates a more stable and functional interaction.

## Supporting information

S1 Fig Cleavage activity of hAgo2. Cleavage of hAgo2 (3.7 μM) was assayed using the following radiolabeled substrates: no guide RNA (negative control), 100 nM guide (p-as2b or p-as2b-FAM) and 2,5 nM target (s2b-^32^P). Samples were collected at 0’, 10’, 30’, 60’ and 120’. Reactions were analyzed by 8% (w/v) PAGE.
(TIF)
