# Supplementary figures and images for: Analysis of AgoshRNA maturation and loading into Ago2
Source: PLoS One. 2017 Aug 15;12(8):e0183269. doi: 10.1371/journal.pone.0183269 (PMC5557517; doi:10.1371/journal.pone.0183269)

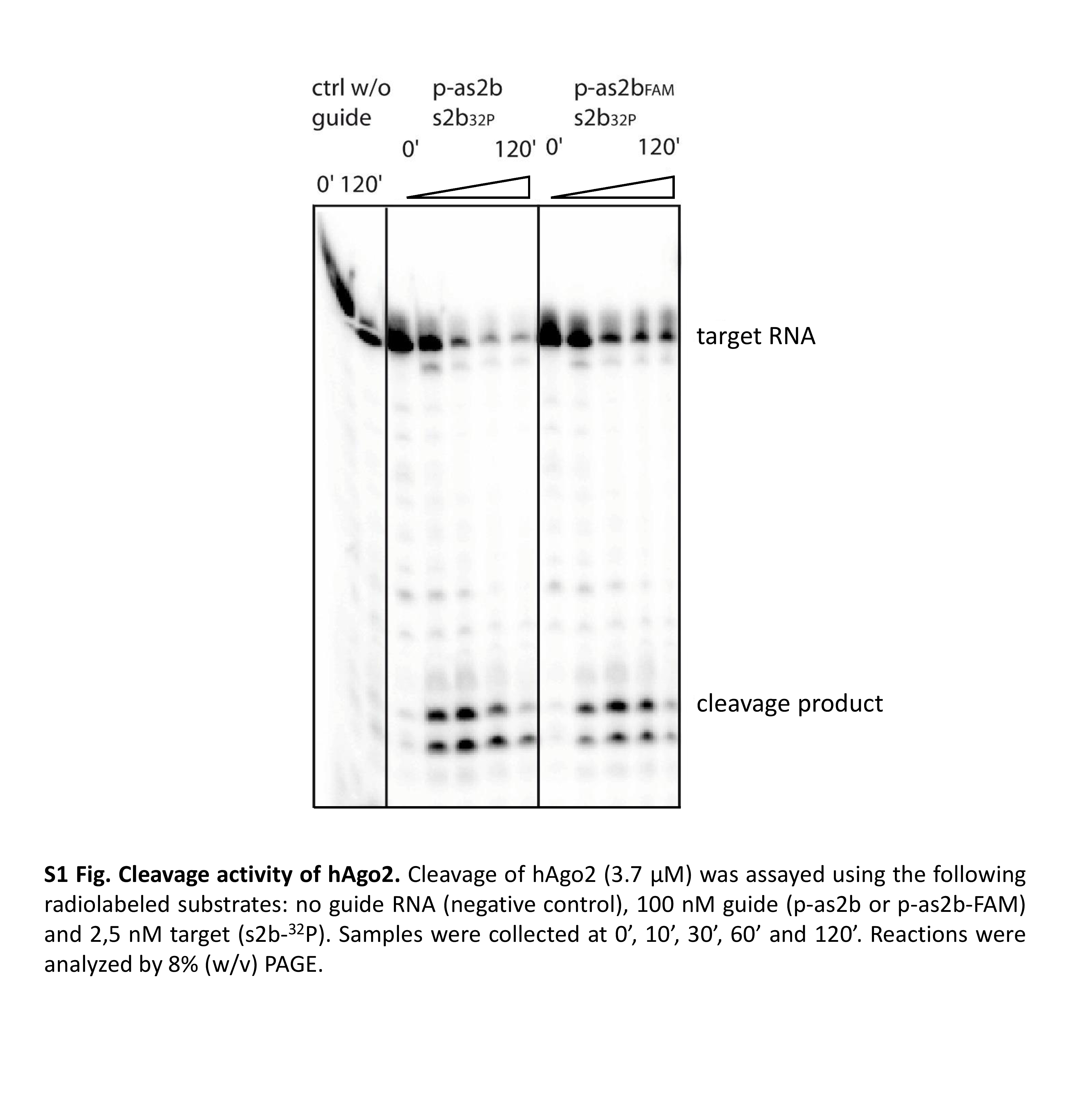

Supplement: S1 Fig — Cleavage of hAgo2 (3.7 μM) was assayed using the following radiolabeled substrates: no guide RNA (negative control), 100 nM guide (p-as2b or p-as2b-FAM) and 2,5 nM target (s2b-32P). Samples were collected at 0’, 10’, 30’, 60’ and 120’. Reactions were analyzed by 8% (w/v) PAGE. (TIF) [file pone.0183269.s001.tif]
